# Vascular remodeling in asthma: from mechanisms to precision medicine

**DOI:** 10.1097/ACI.0000000000001164

**Published:** 2026-05-14

**Authors:** Remo Poto, Rory Chan, Gianluca Lagnese, Andrea Portacci, Gilda Varricchi

**Affiliations:** a Department of Translational Medical Sciences, University of Naples Federico II, Naples, Italy; b University of Dundee, School of Medicine, Dundee, Scotland, UK; c Institute of Respiratory Disease, Department of Translational Biomedicine and Neuroscience, University “Aldo Moro”, Bari; d Center for Basic and Clinical Immunology Research (CISI), University of Naples Federico II, Naples, Italy

**Keywords:** airway remodeling, angiogenesis, asthma, endothelial dysfunction, precision medicine

## Abstract

**Purpose of review:**

Angiogenesis and lymphangiogenesis are increasingly recognized as dynamic components of airway remodeling in asthma, with potential contributions to airway wall thickening, edema, ventilation–perfusion mismatch and fixed airflow limitation. This review updates mechanistic and translational evidence from the most recent literature and positions vascular biology within precision medicine and disease-modification frameworks.

**Recent findings:**

Recent integrative concepts place epithelial barrier dysfunction and alarmin programs upstream of structural disease, providing a biologically plausible bridge between chronic airway injury, innate immune activation and endothelial plasticity across asthma endotypes. Advanced imaging is reshaping phenotyping by enabling non-invasive assessment of structure–function relationships, including airway wall abnormalities and vascular/perfusion signatures relevant to longitudinal monitoring. Long-term data on upstream biologic therapy support sustained clinical benefits over years, strengthening the rationale to test whether vascular abnormalities are modifiable endpoints rather than static consequences of past inflammation.

**Summary:**

Vascular remodeling should be considered a clinically meaningful dimension of asthma heterogeneity that may explain persistent impairment despite inflammatory control. Integrating circulating angiogenesis-related mediators with imaging-derived vascular indices could refine risk stratification, inform treatment selection and accelerate a shift from symptom control toward durable disease modification.

## INTRODUCTION

Airway remodeling is a fundamental determinant of asthma severity, progression and long-term outcomes, extending beyond inflammation to persistent structural alterations of the airway wall [1]. These changes encompass epithelial injury, subepithelial fibrosis, airway smooth muscle hypertrophy and hyperplasia, mucus gland enlargement, and profound modifications of the airway vasculature [2^▪▪^]. 

**Box 1:**
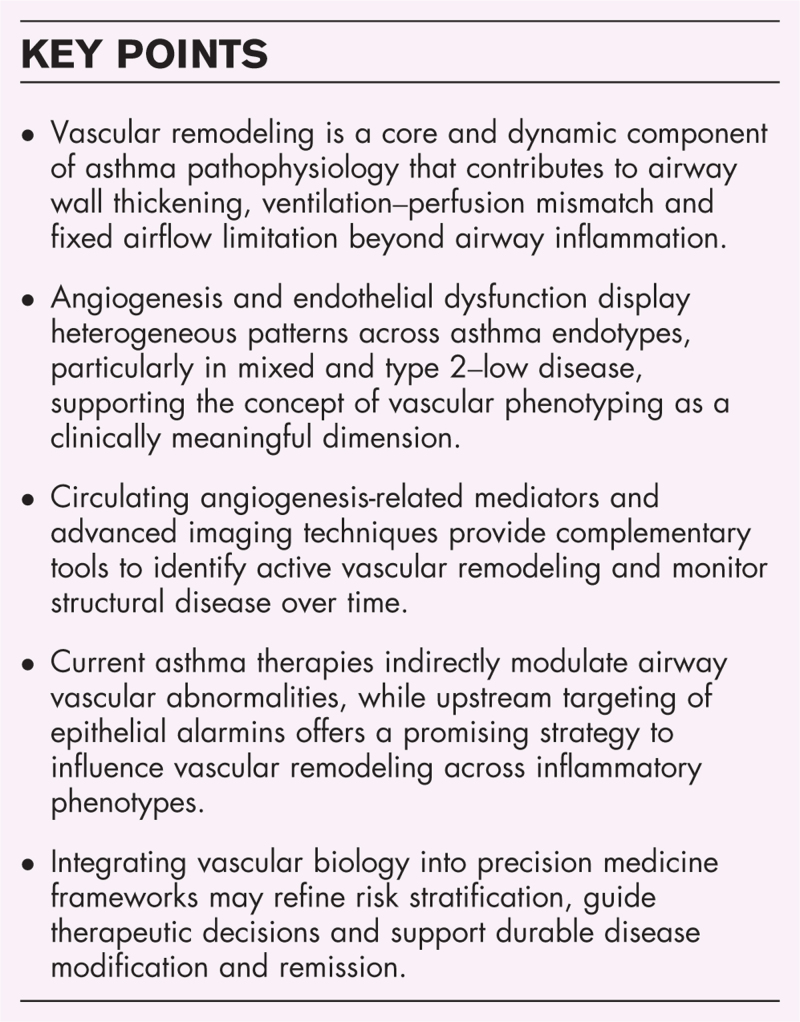
no caption available

While inflammatory pathways have historically dominated mechanistic research and therapeutic development, structural components of asthma have increasingly emerged as independent drivers of disease persistence and functional impairment [3^▪▪^,^4^].

Among the various elements of airway remodeling, vascular alterations have received comparatively limited attention, despite consistent histological evidence of increased microvascular density, enlarged vascular area and enhanced endothelial activation in asthmatic airways [5^▪^]. Importantly, angiogenesis is detectable not only in severe disease but also in mild asthma and in atopic individuals without overt airflow limitation, suggesting that vascular remodeling may accompany early disease development rather than represent a late consequence of chronic inflammation [6]. These observations challenge the traditional view of angiogenesis as a secondary epiphenomenon and support its inclusion among core structural disease mechanisms [7^▪▪^].

Functionally, increased airway vascularity and endothelial permeability contribute directly to airway wall thickening through tissue edema and plasma exudation and indirectly by facilitating inflammatory cell trafficking and mediator diffusion [8^▪▪^,^9^^▪▪^]. Such mechanisms provide a plausible explanation for the frequent dissociation between inflammatory biomarkers and lung function, particularly in patients with persistent airflow limitations despite apparent inflammatory control. Accordingly, vascular remodeling may represent a missing link between inflammation and irreversible structural change [10^▪^].

Over the past decade, advances in imaging, tissue-based analyses and molecular profiling have substantially refined our understanding of airway vascular biology in asthma [11^▪▪^]. Quantitative imaging techniques have revealed heterogeneity in vascular and perfusion abnormalities not captured by spirometry or conventional inflammatory markers. (20,21) In parallel, epithelial stress responses and innate immune pathways, including alarmin signaling, have emerged as upstream regulators of endothelial activation and angiogenic programs across asthma endotypes [12,13].

The advent of biologic therapies targeting type 2 cytokines and, more recently, epithelial alarmins has provided an unprecedented opportunity to interrogate whether airway remodeling, including its vascular component, is modifiable [2^▪▪^,^14^,^15^]. Long-term extension studies and real-world data increasingly support the concept that structural disease components may be dynamic in subsets of patients, raising the possibility that vascular remodeling could represent a treatable and potentially disease-modifying trait [3^▪▪^].

**FIGURE 1:**
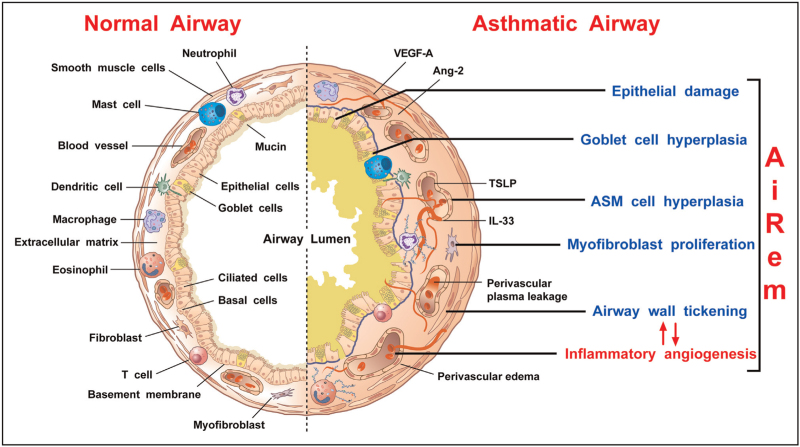
Structural and vascular remodeling in asthma. Schematic cross-sectional representation of a normal (left) and asthmatic (right) bronchial wall. Compared with the normal airway, the asthmatic airway exhibits epithelial damage, goblet cell hyperplasia with mucus accumulation, smooth muscle hypertrophy and hyperplasia, and subepithelial fibrosis contributing to airway wall thickening. Concomitantly, the lamina propria shows increased microvascular density, vessel dilation, endothelial activation and perivascular plasma leakage, consistent with inflammatory angiogenesis. Epithelial-derived alarmins, including TSLP and IL-33, together with angiogenic mediators such as VEGF-A and Ang-2, contribute to the amplification of vascular remodeling, linking immune activation to structural alterations of the airway wall.

In this context, integrating vascular biology into precision medicine frameworks may refine phenotyping, improve risk stratification and inform therapeutic decision-making beyond conventional inflammatory endotypes [7^▪▪^]. Vascular remodeling should therefore be considered an integral component of airway structural disease in asthma, rather than a secondary epiphenomenon (Fig. 1).

This review critically appraises recent advances in the understanding of angiogenesis and lymphangiogenesis in asthma, with a focus on mechanistic insights, structural and imaging correlates, endotype-specific patterns, biomarker development and therapeutic implications, aiming to position vascular remodeling as a clinically meaningful dimension of asthma heterogeneity.

### Angiogenesis and lymphangiogenesis in the asthmatic airway

Angiogenesis and lymphangiogenesis are highly regulated biological processes that remain largely quiescent in adult tissues under physiological conditions, becoming activated primarily during wound healing, inflammation and tissue remodeling [16]. In the lung, vascular homeostasis reflects a delicate balance between pro-angiogenic and anti-angiogenic signals that ensure adequate tissue perfusion while preserving barrier integrity and gas exchange [17].

In asthma, this balance is disrupted, leading to sustained activation of angiogenic pathways within the airway wall [10^▪^]. Histopathological studies consistently demonstrate increased microvascular density, enlarged vascular cross-sectional area and endothelial cell proliferation in the bronchial mucosa of patients with asthma compared with healthy controls [18^▪^]. These changes are detectable across disease severities and are already present in mild asthma, supporting the concept that angiogenesis is an early and intrinsic component of airway remodeling [19^▪^].

Vascular endothelial growth factor (VEGF) represents a central mediator of airway angiogenesis in asthma [20]. Increased VEGF expression has been documented in bronchial biopsies, induced sputum and bronchoalveolar lavage fluid from asthmatic patients, where it correlates with vascular density, airway hyperresponsiveness and disease severity [21]. VEGF promotes endothelial proliferation, migration and survival predominantly through activation of VEGF receptor-2, while also increasing vascular permeability and plasma extravasation [22].

Beyond VEGF, the angiopoietin–Tie2 system plays a crucial role in regulating vascular stability and remodeling in chronic airway disease [23]. Angiopoietin-1 supports endothelial quiescence and vessel maturation, whereas angiopoietin-2 acts as a context-dependent antagonist that destabilizes the endothelium and sensitizes vessels to inflammatory and angiogenic stimuli [24^▪^].

In asthma, increased angiopoietin-2 expression has been associated with endothelial activation, vascular leakiness and more severe disease phenotypes [25].

Angiogenesis in asthma is closely linked to chronic inflammation but cannot be fully explained by inflammatory cell burden alone. Several inflammatory cells, including eosinophils, mast cells and macrophages, directly contribute to angiogenesis through the release of VEGF, angiogenin, basic fibroblast growth factor and matrix-remodeling proteases [23]. These mediators not only promote new vessel formation but also modify the extracellular matrix, creating a permissive microenvironment for sustained vascular remodeling [7^▪▪^].

Lymphangiogenesis represents an additional, often overlooked component of vascular remodeling in asthma [10^▪^]. Lymphatic vessels are essential for maintaining tissue fluid balance and for the clearance of inflammatory mediators and immune cells from the airway wall [26]. Experimental and human studies suggest that altered lymphatic density or function may impair interstitial fluid drainage, thereby perpetuating airway wall edema and chronic inflammation [27].

Importantly, angiogenesis and lymphangiogenesis are not necessarily adaptive responses in asthma. Persistent activation of these pathways may contribute to airway wall thickening, reduced airway compliance and progressive loss of lung function over time [28]. These observations provide a biological rationale for considering vascular remodeling not merely as a consequence of inflammation, but as an active driver of structural disease with direct clinical relevance.

### Structural and imaging evidence of vascular remodeling in asthma

Histological studies consistently show increased microvascular density and endothelial activation in the bronchial mucosa of patients with asthma, changes that correlate with airway hyperresponsiveness and airflow limitation [10^▪^,^29^^▪▪^,^30^^▪▪^].

Computed tomography has enabled in vivo quantification of structural remodeling and provided a bridge between biopsy-defined pathology and clinically actionable phenotypes [11^▪▪^,^31^]. Early helical and multidetector CT studies demonstrated that airway wall thickening is a reproducible feature of asthma and is associated with disease severity and lung function impairment [32,33]. In severe asthma, multidetector CT shows greater airway wall thickness than in mild disease or health [32,33]. Quantitative CT analyses have further demonstrated that airway remodeling and air trapping cluster into distinct imaging phenotypes, supporting the heterogeneity of structural disease in asthma [34,35]. Notably, studies integrating bronchial biopsy data with thoracic quantitative CT suggest that tissue remodeling associates with CT features and functional impairment, supporting an integrated structural framework that is directly relevant to precision medicine [36].

Bronchoscopic imaging has provided complementary *in vivo* information on airway wall structure [37,38]. Endobronchial ultrasound can resolve airway wall layers and has shown increased wall thickness in asthma, with translational value for mechanistic studies and interventional trials [39,40].

Functional imaging is extending structural assessment beyond geometry [41]. Hyperpolarized xenon-129 ventilation MRI can quantify ventilation defects in difficult and severe asthma and has been proposed as a clinically meaningful readout of small-airway obstruction that complements CT-derived remodeling metrics [41,42]. Reproducibility studies across sites support the feasibility of xenon-129 ventilation MRI as a standardized endpoint in severe asthma research, which is essential if imaging is to be adopted as a surrogate marker of disease modification [43].

Collectively, tissue and imaging evidence indicate that vascular remodeling in asthma is measurable, heterogeneous and clinically relevant for precision phenotyping and longitudinal monitoring.

### Cellular drivers and vascular crosstalk: epithelium, innate immunity, and structural cells

**FIGURE 2:**
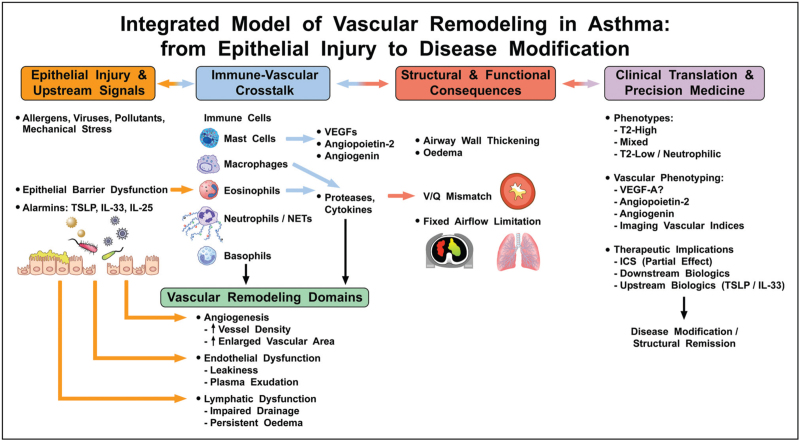
Integrated model of vascular remodeling in asthma. Epithelial injury induced by environmental triggers leads to the release of upstream alarmins, including thymic stromal lymphopoietin (TSLP), interleukin-33 (IL-33) and interleukin-25 (IL-25), which activate innate and adaptive immune pathways and directly modulate endothelial function. Immune–vascular crosstalk involving mast cells, eosinophils, neutrophils, macrophages and structural cells promotes angiogenesis, endothelial destabilization and lymphatic dysfunction through the release of vascular endothelial growth factor (VEGF), angiopoietin-2 and angiogenin. These vascular alterations contribute to airway wall thickening, oedema, ventilation–perfusion mismatch and fixed airflow limitation. Integration of circulating angiogenesis-related biomarkers with advanced imaging enables vascular phenotyping across asthma endotypes and supports the concept of vascular remodeling as a modifiable domain and a potential target for disease modification.

Airway angiogenesis in asthma results from coordinated crosstalk among epithelial cells, immune effectors and structural compartments [7^▪▪^,^44^,^45^,46^▪^]. Accordingly, the airway vasculature should be viewed as a responsive tissue interface that integrates barrier injury, immune activation and mechanical stress into durable structural change [44]. The complex cellular and molecular interactions driving airway vascular remodeling are summarized in Fig. 2.

Epithelial barrier dysfunction is increasingly recognized as an upstream driver of both inflammation and remodeling in asthma [47]. Epithelial cells respond to allergens, pollutants and viral injury by activating stress programs and releasing alarmins that shape downstream immune and structural responses [48]. These epithelial programs can plausibly engage endothelial activation directly through soluble mediators and indirectly by amplifying innate and adaptive immune circuits that are intrinsically pro-angiogenic [49].

Thymic stromal lymphopoietin (TSLP) and interleukin (IL)-33 are particularly relevant in this context because they function upstream of multiple inflammatory endotypes and can bridge epithelial injury to structural remodeling [50,51]. Importantly, both TSLP and IL-33 are expressed not only by epithelial cells but also by endothelial cells, supporting the concept that the airway vasculature actively participates in alarmin-driven inflammatory and remodeling programs rather than serving as a passive downstream target [52^▪^]. Beyond expression, TSLP can directly act on endothelial cells, promoting their activation and functional modulation, thereby reinforcing the bidirectional crosstalk between epithelial stress responses and vascular remodeling [53^▪^].

IL-33 signaling is mechanistically linked to airway remodeling programs and may contribute to endothelial activation and vascular leakiness through innate immune amplification even when downstream type 2 markers are not dominant [54]. This is clinically meaningful because it provides a framework to explain persistent impairment in patients whose inflammatory biomarkers suggest “control” yet whose structural disease remains active [7^▪▪^].

Neutrophils represent a key cellular node connecting innate inflammation to vascular dysfunction in severe asthma [22]. Environmental exposures can instruct recruited lung neutrophils toward asthma-promoting phenotypes, supporting the idea that neutrophil-driven mechanisms are not merely “bystanders” but can shape disease initiation and persistence [55]. Activated neutrophils have been shown to release VEGF-A in response to inflammatory stimuli, and this neutrophil-derived VEGF-A can drive endothelial permeability and angiogenic responses, highlighting a direct link between innate immune activation and vascular remodeling [56].

Neutrophil extracellular traps (NETs) provide an additional mechanistic layer, as NET formation is increasingly recognized as a potent amplifier of tissue injury and immune activation across diseases [57]. In airway disease, NETs can intensify epithelial stress responses and propagate inflammatory circuits through pattern-recognition pathways, creating a feed-forward loop that is compatible with sustained endothelial activation and pathological angiogenesis [58]. Beyond tissue injury, NETs have been shown to directly promote angiogenesis and endothelial activation, supporting their role as structural modifiers in neutrophil-dominant and mixed asthma phenotypes [22].

This NET-centered view supports a biologically plausible link between neutrophilic inflammation, endothelial dysfunction and the heterogeneity of remodeling trajectories observed in severe asthma [58].

Mast cells represent another critical link between inflammation and microvascular remodeling because of their strategic localization within airway smooth muscle bundles and perivascular regions [59]. Mast cell-derived mediators can promote angiogenesis directly, and protease-driven matrix remodeling can liberate growth factors and alter the biomechanical microenvironment that supports vessel growth and leakiness [60]. This spatially constrained amplification may be particularly relevant in severe asthma, where mast cell–smooth muscle interactions are prominent and could influence local vascular remodeling within the airway wall [61]. IL-33 has been shown to induce the release of VEGF-A and VEGF-C from human lung mast cells, and to potentiate the production of angiogenic and lymphangiogenic mediators upon immunologic activation, thereby reinforcing the role of mast cells in airway vascular remodeling [62].

Macrophages, fibroblasts and airway smooth muscle cells further contribute to angiogenic signaling by producing growth factors and remodeling extracellular matrix, thereby shaping endothelial behavior in a context-dependent manner [5^▪^]. Notably, TSLP enhances VEGF-A production by human lung macrophages and eosinophils, providing a mechanistic link between epithelial alarmin activation and amplification of angiogenic signaling within the airway microenvironment [5^▪^]. Human lung-resident macrophages have been shown to produce VEGF-A and -C and angiopoietins, supporting their contribution to angiogenic and lymphangiogenic remodeling [63–65].

The convergence of epithelial stress signaling, innate immune activation and mesenchymal remodeling supports a model in which vascular remodeling is a composite structural trait, potentially dissociable from any single inflammatory biomarker [20]. This has direct implications for precision medicine because it argues for integrating vascular indices with inflammatory and functional markers to capture the “structural burden” that determines long-term outcomes [66].

### Angiogenesis across asthma endotypes: towards vascular phenotyping and precision medicine

Airway vascular remodeling is not uniform across asthma and should be interpreted as an endotype-modifying dimension that intersects, but does not overlap perfectly with, canonical inflammatory stratification [46^▪^]. The current framework of severe asthma recognizes type 2–high, type 2–low and mixed endotypes, and explicitly acknowledges that remodeling trajectories can be partially dissociable from inflammatory biomarkers [67]. This conceptual separation is clinically important because it creates a rationale for “vascular phenotyping” when symptoms, exacerbations or lung function impairment persist despite apparently adequate suppression of airway inflammation [68].

In type 2-high asthma, proangiogenic signals are often interpreted as downstream consequences of eosinophilic inflammation and epithelial activation, and vascular abnormalities may improve with anti–type 2 strategies in parallel with clinical response [7^▪▪^]. However, even within type 2–high disease, structural abnormalities can be disproportionate to inflammatory readouts, suggesting that vascular remodeling can acquire partial autonomy through tissue memory mechanisms, matrix remodeling and endothelial reprogramming [67].

In non-type 2 and type 2-low asthma, angiogenesis may be driven by alternative inflammatory programs, including type 1 and type 17 pathways, chronic epithelial stress responses and innate immune activation [68]. Type 2-low asthma remains a heterogeneous umbrella that includes neutrophilic, paucigranulocytic and type 1 or type 17-skewed patterns, each of which may plausibly engage distinct vascular mechanisms [69]. Absence of T2 biomarkers does not exclude active structural disease, and vascular remodeling may represent a treatable trait even in apparently non-eosinophilic phenotypes [70].

Recent evidence in severe mixed or neutrophilic asthma suggests that vascular remodeling can be accompanied by an overexpression of upstream epithelial pathways and angiogenesis-related mediators, supporting a mechanistic bridge between epithelial injury, innate inflammation and endothelial activation [71^▪▪^]. From a biomarker perspective, angiopoietin-2 has been associated with severe refractory asthma and exacerbation burden, supporting the concept that endothelial destabilization and leakiness can track clinical severity [72^▪^]. Similarly, contemporary biomarker-focused syntheses highlight angiogenesis-related mediators as candidate additions to current algorithms, particularly for patients with persistent symptoms or exacerbations despite high-intensity anti-inflammatory therapy [73]. These observations encourage an integrated approach in which vascular biomarkers are interpreted alongside inflammatory markers, lung function, and imaging phenotypes, rather than as stand-alone classifiers.

The rise of broad-acting and upstream biologics further increases the practical relevance of vascular phenotyping [74]. In non–type 2 severe asthma, mechanistic reviews emphasize the need for multidimensional assessment and for endpoints that capture structural disease domains, providing a conceptual basis to test whether upstream interventions can modulate vascular remodeling across endotypes [68]. In parallel, treatable-traits frameworks increasingly propose that asthma management should target measurable disease components beyond inflammation alone, a strategy that naturally accommodates vascular remodeling when supported by biomarkers or imaging [75].

Collectively, these data support a pragmatic hypothesis: vascular remodeling may define an actionable dimension of asthma heterogeneity that improves the alignment between mechanisms and clinical decisions, particularly in severe, mixed, and type 2–low disease. Operationalizing this concept requires validated biomarker panels and imaging-derived vascular indices, together with longitudinal studies linking vascular change to outcomes that matter, including exacerbations, lung function decline and remission [75]. If confirmed, vascular phenotyping could help transform asthma precision medicine from an inflammation-centered paradigm to a multidomain model that explicitly incorporates structural stabilization as a therapeutic goal.

### Biomarkers and clinical surrogates of vascular remodeling: from blood to imaging and composite endpoints

A major barrier to integrating vascular remodeling into precision medicine is the lack of validated biomarkers reflecting endothelial activation and angiogenic activity [7^▪▪^].

### Circulating biomarkers of airway angiogenesis: promise and pitfalls

VEGF is a prototypical angiogenic mediator in asthma but circulating VEGF lacks disease specificity because it is influenced by systemic inflammation, metabolic factors and comorbidities [18^▪^]. It is therefore unlikely to serve as a stand-alone stratification marker, although it remains informative within mechanistic biomarker panels. [30^▪▪^]. Recently, attention has shifted toward mediators that better capture endothelial destabilization and leakiness, which are closer to the functional consequences of vascular remodeling than vessel number alone [24^▪^]. Angiopoietin-2 is attractive in this regard because it signals vascular instability and cooperates with inflammatory cues to amplify permeability and pathological remodeling [25]. Clinical work supports associations between angiopoietin-2 and severe or refractory asthma, strengthening the hypothesis that endothelial destabilization tracks clinically meaningful disease burden [72^▪^].

Angiogenin has emerged as a candidate marker of a “vascular-dominant” phenotype [71^▪▪^]. However, the clinical utility of angiogenin and related mediators will depend on standardization of assays, definition of decision thresholds and validation across independent, longitudinal cohorts [73].

### Imaging-derived biomarkers: turning structure into longitudinal metrics

Imaging offers a parallel route to vascular phenotyping by capturing structural and functional consequences of vascular remodeling *in vivo*[11^▪▪^]. Quantitative CT is already a robust platform for measuring airway wall thickness, air trapping and remodeling clusters, providing structural context for vascular hypotheses [32,34,76]. Importantly, studies linking bronchial biopsy remodeling to thoracic quantitative CT support the biological validity of imaging as a surrogate endpoint for structural disease domains [36]. This is a key methodological advance because vascular remodeling may be difficult to monitor repeatedly with invasive tissue sampling in routine care or long trials [37].

Beyond airway geometry, functional vascular information is increasingly accessible through advanced imaging [11^▪▪^]. Dual-energy CT and related approaches may capture perfusion abnormalities relevant to vascular dysfunction, although their longitudinal role remains to be defined [77–81]. Hyperpolarized xenon-129 MRI further captures ventilation heterogeneity in difficult and severe asthma and can be paired conceptually with perfusion indices to interrogate ventilation–perfusion mismatch at high resolution [41]. The feasibility and reproducibility of these approaches across sites strengthens their candidacy for inclusion as endpoints in disease-modification trials [43].

### Composite endpoints and trial-readiness: where precision medicine can realistically go next

For clinical translation, combining circulating angiogenesis mediators with imaging-derived indices may capture both biological activity and structural consequences of vascular remodeling, consistent with multidomain precision-medicine approaches [68]. In practice, composite frameworks integrating angiopoietin-2 or angiogenin with CT remodeling phenotypes and perfusion metrics may help identify patients with active vascular remodeling despite controlled inflammation and may provide more suitable endpoints for trials testing structural disease modification. Biomarker-imaging integration may also support future definitions of structural remission.

### Therapeutic implications: from indirect modulation of angiogenesis to vascular normalization

Current asthma therapies were not developed to directly target angiogenesis; nevertheless, inhaled glucocorticoids (ICS) can exert indirect effects on the airway vasculature that may influence structural disease trajectories [29^▪▪^]. ICS reduce airway vascularity and permeability primarily through suppression of pro-angiogenic mediators, including VEGF, and by restoring epithelial barrier integrity [29^▪▪^]. However, ICS incompletely reverse established vascular remodeling, particularly in severe disease, suggesting that vascular abnormalities may become partially corticosteroid-insensitive over time [29^▪▪^].

Biologic therapies targeting type 2 inflammation have further clarified the dissociation between inflammatory control and structural normalization. Anti-IL-5 and anti-IL-5 receptor alpha therapies improve exacerbation rates and eosinophilic inflammation but show variable effects on airway remodeling, indicating that suppression of eosinophils alone is insufficient to uniformly modulate vascular pathology [3^▪▪^,^82^]. Similarly, IL-4Rα blockade improves clinical outcomes and mucus-related imaging features, yet persistent airway wall thickening and perfusion abnormalities have been reported in subsets of treated patients [83].

These observations have redirected attention toward upstream epithelial and innate immune pathways that sit closer to the initiation of angiogenic programs [84]. Targeting TSLP is particularly attractive because TSLP integrates epithelial injury, innate immune activation and downstream inflammatory cascades across asthma endotypes [85]. Long-term extension studies of tezepelumab demonstrate sustained clinical benefit over years, providing a necessary prerequisite to interrogate whether prolonged upstream inhibition can influence structural domains, including vascular remodeling [5^▪^,^86^,^87^].

Mechanistic trials further support this rationale. In the CASCADE study, tezepelumab reduced airway inflammatory cell infiltration and hyperresponsiveness, although effects on classical remodeling endpoints were modest over the study duration, highlighting the need for longer follow-up and more sensitive vascular readouts [88].

The concept of vascular normalization, rather than indiscriminate anti-angiogenesis, provides a clinically relevant therapeutic framework. Vascular normalization aims to restore endothelial integrity, reduce pathological permeability and re-establish functional microvascular architecture, thereby improving tissue oxygenation and reducing edema without impairing physiological repair mechanisms [89].

From a clinical trial perspective, incorporating vascular endpoints is increasingly feasible. Quantitative CT metrics, perfusion imaging and circulating endothelial markers could be used to better characterize patients with asthma, monitor treatment response and distinguish disease modification from symptomatic improvement [73]. Such an approach is particularly relevant for type 2–low and mixed severe asthma, where therapeutic options remain limited and where vascular remodeling may represent a shared, targetable structural trait [68,71^▪▪^].

Ultimately, integrating vascular biology into therapeutic decision-making challenges the current inflammation-centric paradigm and supports a shift toward multidomain treatment goals that explicitly include structural stabilization [75]. If validated, this strategy could refine patient selection for upstream biologics, guide treatment duration and contribute to durable disease modification rather than symptomatic control [90].

## CONCLUSION

The growing body of evidence reviewed here supports the view that vascular remodeling is not a passive epiphenomenon of airway inflammation, but a biologically active and clinically relevant component of asthma pathophysiology [10^▪^,46^▪^,^91^].

Importantly, vascular abnormalities may persist despite apparent inflammatory control, contributing to airflow limitation, symptom burden and exacerbation risk even in patients treated with high-intensity anti-inflammatory regimens [7^▪▪^].

From a conceptual standpoint, these observations challenge the traditional inflammation-centric model of asthma management and argue for a multidomain approach that explicitly incorporates vascular biology. In this framework, angiogenesis and endothelial dysfunction represent modifiable traits that may influence disease trajectory independently of classical inflammatory markers. Recognizing vascular remodeling as a treatable dimension supports multidomain precision medicine [75].

Upstream targeting of epithelial and innate immune pathways offers a particularly promising avenue to test these concepts [92^▪▪^].

By acting closer to the initiating events of airway injury and stress responses, therapies targeting TSLP or IL-33 may exert broader effects on downstream inflammatory and structural programs, including vascular remodeling [47]. Experimental and clinical data indicate that TSLP blockade can reduce VEGF-A expression, further supporting the hypothesis that upstream inhibition may indirectly modulate angiogenic pathways and vascular remodeling [5^▪^,^93^].

In this context, vascular normalization emerges as a clinically meaningful therapeutic goal. Rather than suppressing angiogenesis indiscriminately, restoring endothelial integrity, reducing pathological permeability and re-establishing functional microvascular architecture may improve airway mechanics and tissue homeostasis without compromising physiological repair. This paradigm is particularly relevant for severe, mixed and type 2–low asthma, where conventional anti-inflammatory strategies often fail to fully restore lung function [71^▪▪^].

Looking forward, the integration of circulating angiogenesis-related biomarkers with quantitative imaging holds promise for operationalizing vascular phenotyping in both trials and clinical practice [73].

Such composite approaches could identify patients with active vascular remodeling, monitor response to upstream therapies and help distinguish symptomatic improvement from genuine structural stabilization.

## Acknowledgements

*The authors thank Dr. Gjada Criscuolo for her excellent managerial assistance.*

### Financial support and sponsorship

*The authors declare that no funding was received for this research.*

*Author contributions: RP, RC and GV wrote the first draft of the manuscript. R.P., R.C., G.L., A.P., G.V. reviewed and modified the manuscript. All authors contributed to the refinement of the final version and agreed to the decision to submit for publication.*

*Data availability statement: Data sharing is not applicable to this article as no datasets were generated or analyzed during the current study.*

### Conflicts of interest

*R.P. reports personal fees (talks) from AstraZeneca and GSK.*

*R.C. reports institutional grants from Chiesi, AstraZeneca and GlaxoSmithKline for setting up and chairing the Scottish Airways Research Network; serving on an advisory board for AstraZeneca; personal fees (talks and drafting educational material) from AstraZeneca, personal fees (talks) from Chiesi, personal fees (talks) from Thorasys and personal fees (drafting educational material) from Vitalograph; support attending meetings from AstraZeneca, Chiesi, NIOX, Sanofi-Regeneron and Vitalograph.*

*G.L. has no conflict of interests to declare.*

*A.P. reports payment or honoraria for lectures, presentations, speakers’ bureaus, manuscript writing, or educational events from Astrazeneca, GlaxoSmithKline, Chiesi, Sanofi. Remo Poto reported personal fees from Astrazeneca and GSK.*

*G.V. reports research support from AstraZeneca. S.E.B. reports consulting for ALK Abellò.*

## REFERENCES AND RECOMMENDED READING

Papers of particular interest, published within the annual period of review, have been highlighted as:

▪ of special interest
▪▪ of outstanding interest
